# Classification and Incidence of Heterotopic Ossifications in Relation to NSAID Prophylaxis after Elbow Trauma

**DOI:** 10.3390/jcm13030667

**Published:** 2024-01-24

**Authors:** Diane Leyder, Stefan Döbele, Christian Konrads, Tina Histing, Cornelius S. Fischer, Marc-Daniel Ahrend, Patrick Ziegler

**Affiliations:** 1 Department of Traumatology and Reconstructive Surgery, BG Unfallklinik Tübingen, Eberhard Karls University of Tübingen, 72074 Tübingen, Germany; 2 Medical Faculty, University of Tübingen, 72074 Tübingen, Germany; christian.konrads@gmail.com (C.K.);; 3 Department of Orthopaedics and Traumatology, Helios Hanseatic Hospital Stralsund, 18435 Stralsund, Germany; 4 Department of Orthopaedics and Trauma Surgery, Klinik Gut, 7500 St. Moritz, Switzerland

**Keywords:** elbow trauma, heterotopic ossifications, classification, stiff elbow, HO prophylaxis

## Abstract

Heterotopic ossification (HO) after elbow trauma can be responsible for significant motion restrictions. The study’s primary aim was to develop a new X-ray-based classification for HO of the elbow. This retrospective study analyzed elbow injury radiographs from 138 patients aged 6–85 years (mean 45.9 ± 18) who underwent operative treatment. The new classification was applied at 6 weeks, 12 weeks, and 6 months postoperatively. The severity of HO was graded from 0 to 4 and localization was defined as r (radial), p (posterior), u (ulnar) or a (anterior) by two observers. The patients were categorized based on injury location and use of non-steroidal anti-inflammatory drugs (NSAIDs) for HO prophylaxis. The correlations between the generated data sets were analyzed using Chi-square tests (χ^2^) with a significance level of *p* < 0.05. The inter- and intraobserver reliability was assessed using Cohen’s Kappa. In 50.7% of the evaluated X-rays, the formation of HO could be detected after 12 weeks, and in 60% after 6 months. The analysis showed a significant correlation between the injury’s location and the HO’s location after 12 weeks (*p* = 0.003). The use of an NSAID prophylaxis did not show a significant correlation with the severity of HO. The classification showed nearly perfect inter- (κ = 0.951, *p* < 0.001) and intrareliability (κ = 0.946, *p* < 0.001) according to the criteria of Landis and Koch. Based on the presented classification, the dimension and localization of HO in the X-ray image can be described in more detail compared to previously established classifications and, thus, can increase the comparability of results across studies.

## 1. Introduction

Heterotopic ossifications (HO) can occur as a complication after elbow injuries. New lamellar bone is formed in soft tissue, which can occur due to an accompanying inflammatory reaction to a trauma [1,2,3]. Severe forms in the joint area can lead to considerable functional restrictions [4,5,6].

The prevalence of HO shows a wide variance of 10–20% after traumatic brain or spinal cord injury; 20% after forearm fractures; up to 52% after femur fractures, total hip arthroplasty or acetabular fractures; and up to 60% after severe burns [7]. HO’s pathophysiology is not yet fully understood, but tissues prone to HO have excessive inflammatory responses to injury. Recent studies show mesenchymal stem cells and their increased bone morphogenic protein (BMP) activity lead to vascular proliferation and bone formation in HO [8].

The incidence of HO at the elbow joint varies significantly. Douglas et al. reported that 35% of patients required surgical intervention of more severe ossifications after distal intra-articular humerus fractures, while 26% needed the same procedure after elbow dislocation fractures [9]. Liu et al. conducted a systematic review involving over 2000 patients and found that 10% developed radiologically visible HO after total elbow arthroplasty [10]. Wahl et al. investigated the incidence and location of HO after elbow injuries and found that almost all patients developed HO, which could be statistically linked to the injury site [11].

There are various ways to clinically measure the stiffness of an elbow joint. Morrey’s scoring system divides elbow stiffness into three categories: intrinsic, extrinsic, and combined stiffness [12]. Another scoring system, developed by Kay, classifies elbow stiffness based on the involved anatomical structure, which could be soft tissue, bony stiffness, or a combination of both [13]. In Kay’s system, HO refers to bony or combined elbow stiffness.

To radiologically define elbow stiffness and determine the presence of HO, two classifications are used: Brooker’s classification was originally designed for the hip joint and is based on X-ray images in one plane. It presents five categories, from the absence of HO to a bone brace [14]. The Hastings and Graham classification was designed for the elbow. In this classification, a distinction between the presence or absence of HO is made with focus on the functional limitations [15]. However, neither of these classifications describe the localization of ossifications. This is especially relevant in the case of elbow joint ossifications, as their location can be used to predict the extent of elbow functional limitation and can also aid in planning revision surgery or radiotherapy. Furthermore, utilizing standardized nomenclature can offer several benefits for the assessment of HO and may assist in optimizing and standardizing therapy options [11]. 

Therapeutic options for expired HO are limited to surgical excision, which often has unsatisfactory results [16,17]. Early detection and prevention of HO are therefore the priority. Non-steroidal anti-inflammatory drugs (NSAIDs) and radiation are most commonly used to prevent HO [18]. Several systematic reviews and meta-analyses have shown that NSAIDs can prevent heterotopic ossification (HO), particularly after total hip arthroplasty [19,20,21]. NSAIDs work by inhibiting the differentiation of mesenchymal stem cells, which are the precursor cells of osteoblasts, and by reducing the production of pro-inflammatory prostaglandins [22]. However, despite ongoing research, there is significant inconsistency in the available evidence regarding their effectiveness after elbow trauma [22,23,24]. 

This study presents a novel radiographic classification system that precisely describes the degree and location of HO, thereby improving objectivity and functional assessment. We aim to demonstrate, based on our hypothesis, that HO occurs at the same site as the primary injury. Additionally, we aim to establish a correlation between the use of ossification prophylaxis and the development of HO in our cohort.

## 2. Materials and Methods

### 2.1. Patient Collective and Study Design

**Figure 1:**
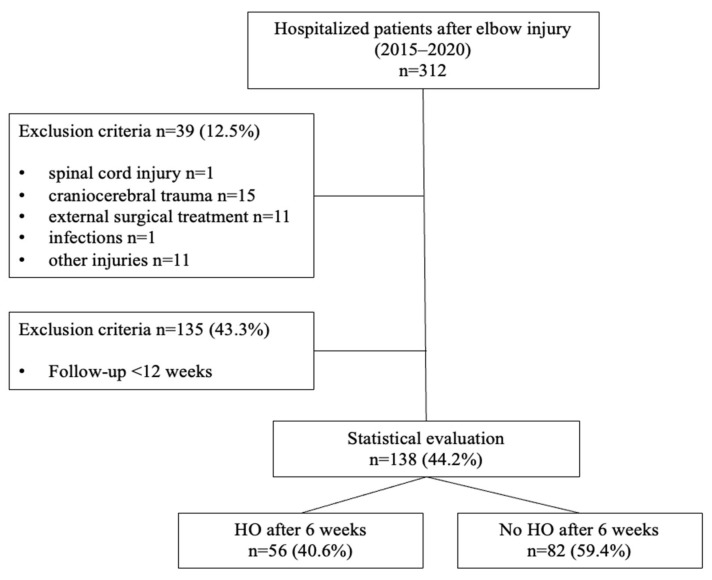
Flowchart of analyzed patients.

This retrospective study was approved by the local ethics committee. The records of 273 patients with surgically treated elbow injuries between January 2015 and December 2020 were analyzed based on inclusion and exclusion criteria. Of the total number of patients considered, 135 were disqualified due to reasons such as spinal cord injury (*n* = 1), traumatic brain injury (*n* = 15), receiving treatment from a different hospital (*n* = 11), infections (*n* = 1), or a follow-up period of less than 12 weeks (*n* = 135). Consequently, 138 patients were deemed eligible and included in the study. Clinical and radiological follow-up exams were conducted at 6 (*n* = 138) and 12 weeks (*n* = 138) as well as 6 months (*n* = 75) post-surgery. The flowchart of patients is shown in Figure 1.

### 2.2. Radiographic Assessment

The analysis of the radiographs was performed initially by a single person using the Xero Universal Viewer software Version 1.0.0.R812 (Xero Universal Viewer, Agfa Health Care Corp. Greenville, SC, USA). For this purpose, standard X-rays of the elbow in two planes (anterior to posterior (AP) and lateral radiographs) were performed two days after the initial surgery. Any preexisting bone avulsions, osteophytes, or ossifications were defined. Follow-up radiographs were realized after six and twelve weeks (*n* = 138), as well as after six months (*n* = 75). After one year, two surgeons in different stages of training reviewed all X-ray images again, blinded to the first interpretation. The images were then analyzed and classified according to the following classification based on the apparent HO.

### 2.3. Classification

For the new classification (Table 1), the localization of HO was defined as follows: radial (r), posterior (p), ulnar (u), and anterior (a). The severity of HO was graded from 0 (no ossifications) to 4 (synostosis). The largest visible HO in terms of length was considered. The classification was determined as follows: 0 means no HO; 1 means visible HO, which is smaller in size than the diameter of the radial head; 2 means a larger diameter than the radial head; 3 indicates a brace formation from the humerus to the forearm; and 4 describes the synostosis radio-ulnar. 

**Figure 2:**
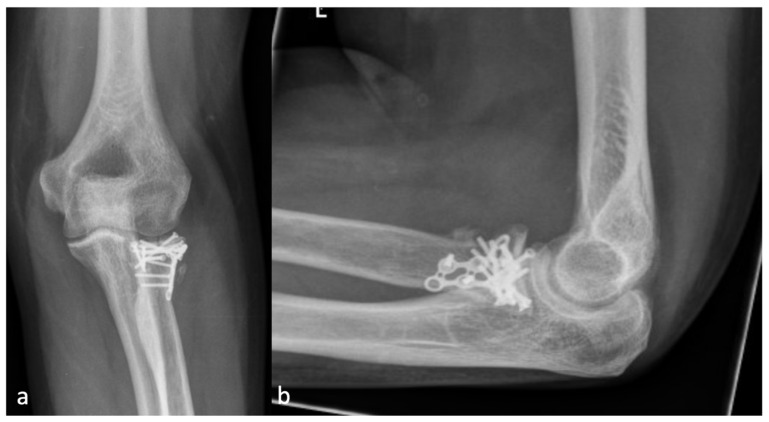
(**a**) The AP and (**b**) the lateral X-ray with a radial HO smaller than the diameter of the radial head, which represents an HO 1 r.

**Figure 3:**
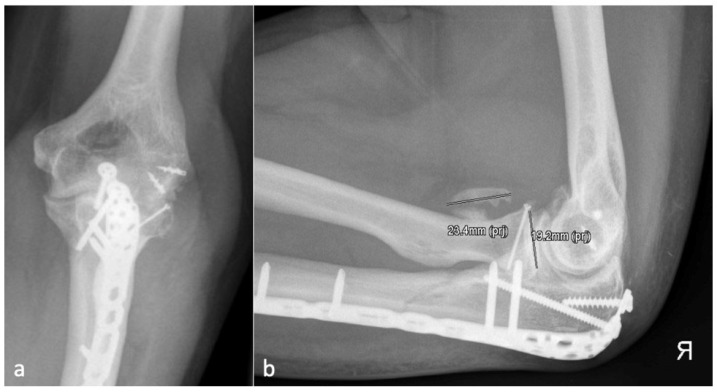
(**a**) shows the AP and (**b**) shows the lateral X-ray with an anterior HO (23.4 mm) which is larger than the diameter of the radial head (19.2 mm) and represents an HO 2a.

**Figure 4:**
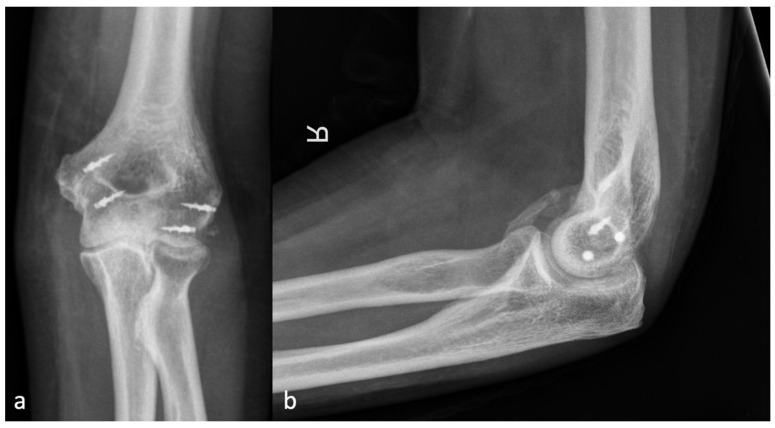
(**a**) shows the AP and (**b**) shows the lateral X-ray with an anterior HO forming a brace from the humerus to the forearm, representing an HO 3a.

**Figure 5:**
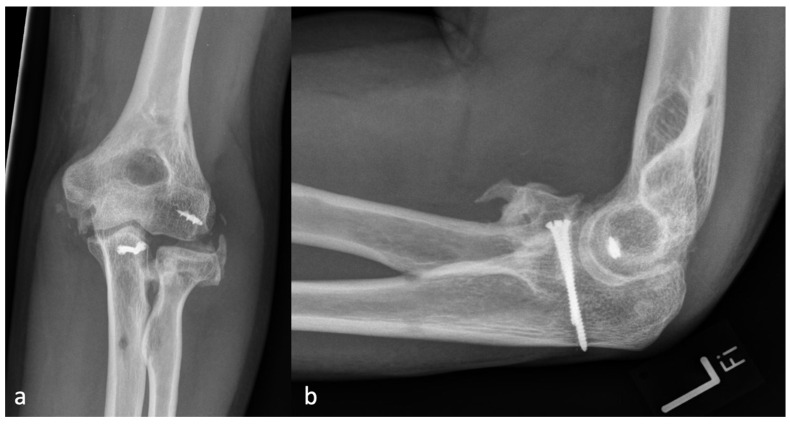
(**a**) shows the AP and (**b**) shows the lateral X-ray with a brace formation radio-ulnar and represents an HO 4.

Since regular radiographs usually do not have a reference sphere, the diameter of the radial head was defined as the cutoff size between severity grades one and two. Thus, the classification is independent of a reference sphere, and the size ratio from the examined elbow is included. Examples of each grade of severity are shown in Figure 2, Figure 3, Figure 4 and Figure 5. 

### 2.4. Further Parameters

The study included a total of 138 patients who were categorized based on their age, sex, and type of injury. We verified the use of ossification prophylaxis by checking the hospital medication records and discharge letters. Ossification prophylaxis was considered to be taken if prescribed for 14 days. Additionally, we determined the location of each injury through a review of the initial X-ray and MRI images taken after the injury.

Five categories of injury were defined:

- Lateral: radial head fracture, LCL tear, lateral condyle fractures of the humerus.
- Ventral: Coronoid fractures.
- Medial: MCL tear, medial condyle fractures of the humerus.
- Dorsal: Olecranon fractures.
- Multilateral: Distal humerus fractures (AO C 1–3), Monteggia fractures, Monteggia-like-lesions, LCL, and MCL rupture at the same time.

### 2.5. Statistical Analysis

Descriptive statistics were performed using absolute and relative frequencies (*n* (%)) or mean ± standard deviation. The incidence of HO was measured after 6 weeks, 12 weeks, and 6 months. The incidence of the injury location at the elbow and the injured structures was calculated. 

This study used Cohen’s Kappa coefficient κ to determine the intra- and interobserver reliability of HO severity and localization. Based on Landis and Koch’s method, values were categorized as ≤0 (no agreement), 0.01–0.20 (none to slight), 0.21–0.40 (fair), 0.41–0.60 (moderate), 0.61–0.80 (substantial), and 0.81–1.00 (almost perfect agreement) [25].

To examine the correlation between HO severity and the use of ossification prophylaxis and between the location of the injury and the developed HO, four-field tables were implemented to present the frequencies of the associations, and significance calculation was performed using the Chi-squared test (χ^2^). A significance level with *p* < 0.05 was defined. The statistical analysis of the collected data was performed using a statistical program (SPSS Statistics^®^, IBM, Armonk, NY, USA, version 28).

## 3. Results

### 3.1. Descriptive Statistics

Among the 138 patients included, 73 (52.9%) were male patients and 65 (47.1%) were female patients. The mean age at trauma was 45.9 ± 18 years (6 to 85 years). The radial head was affected in 61 (44.2%), the coronoid in 36 (26.1%), the olecranon in 22 (15.9%), and the distal humerus in 34 (24.6%) cases. Regarding ligament injuries, the lateral collateral ligament was considered unstable in 33 (23.9%), the lateral and medial collateral ligaments in 38 (27.5%), and the medial collateral ligament alone in three cases (2.2%).

Of the total number of patients under observation, it was noted that a majority of them (102 (73.9%) patients) received prophylaxis for ossification for 14 days, while the remaining 36 (26.1%) did not receive any NSAID prophylaxis. Among the patients who received prophylaxis, 47 (33.9%) were given Indomethacin (100 mg/day), 53 (64.5%) were given Ibuprofen (800–1800 mg/day), and 1 patient each was given Etoricoxib (90 mg/day) or Celecoxib (400 mg/day).

The incidence of HO after 6 weeks (5.8 ± 2.3 weeks) was 40.6% (Grade I 35.5%, Grade II 4.3% and Grade III 0.7%), after 12 weeks (16.4 ± 10.9 weeks) 50.7% (Grade I 41.3%, Grade II 6.5% and Grade III 2.9%), and after 6 months (29.9 ± 10.9 weeks) 60.0% (Grade I 40.0%, Grade II 12.0%, Grade III 2.7%, and Grade IV 5.3%). Details regarding the location and severity of HO are summarized in Table 2. 

### 3.2. Relationship between Injury Localization and the Localization of HO

The study found a significant relation (χ^2^ = 28.3, *p* = 0.005) between the location of the injury (lateral, posterior, medial, anterior, multilateral) and the location of a HO (radial, posterior, ulnar, anterior) after 6 weeks. The correlation remained significant even after 12 weeks (χ^2^ = 25.5, *p* = 0.003). However, after 6 months, the correlation was no longer significant (χ^2^ = 9.1, *p* = 0.421).

### 3.3. Correlation between the Application of Ossification Prophylaxis and the Severity of HO

After 6 weeks, we found no significant correlation (χ^2^ = 1.0, *p* = 0.797) between the severity of HO and the application of ossification prophylaxis. The same results were observed after 12 weeks (χ^2^ = 0.9, *p* = 0.876) and after 6 months (χ^2^ = 5.5, *p* = 0.244) in this cohort. After comparing the two most commonly used methods for preventing ossification, Indomethacin and Ibuprofen, it was found that there was no significant correlation between the severity of HO and the medication taken. This was observed after 6 weeks (χ^2^ = 4.7, *p* = 0.197), after 12 weeks (χ^2^ = 0.9, *p* = 0.819), and after 6 months (χ^2^ = 4.3, *p* = 0.363).

### 3.4. Intra- and Interobserver Reliability 

The level of agreement between raters (interreliability) was found to be almost perfect in both the categorization of the severe gradient (κ = 0.951, *p* < 0.001) and the classification of the localization of HO (κ = 0.953, *p* < 0.001). Similarly, the level of agreement within the same rater (intrareliability) was almost perfect for both the severity grade (κ = 0.946, *p* < 0.001) and the localization (κ = 0.949, *p* < 0.001) of HO.

## 4. Discussion

In the present study, the formation and extent of ossifications at the elbow joint were investigated based on radiological signs concerning the injury pattern and localization. Based on the analyzed data, a classification for HO at the elbow was developed. The goal of the present study was to simplify and standardize the classification of HO around the elbow.

### 4.1. Classification

The aim was to develop a classification that is easy to apply. The severity of HO was divided into five different categories (0–4). Furthermore, the localization of the ossification could be documented by the addition of the letters (anterior, posterior, radial, ulnar). This differs from existing classifications, such as the Hastings and Graham classification, which focuses predominantly on the restriction of movement [15]. The inter- and intrareliability for both the severity level and localization of HO classification was almost perfect. Whether the proposed classification can be easily applied in a clinical daily routine needs to be shown by further studies and regular applications in clinical practice.

### 4.2. Incidences of HO

The incidence of HO after surgically treated elbow injuries in our collective (up to 60%) is much higher than the average values presented in the literature. For example, Herman et al. described an incidence of 28.7% after surgically treated elbow fractures [26]. Foruria et al. studied distal humerus fractures that were primarily treated via osteosynthesis; here, 42% showed HO at 12 weeks [27]. Hong et al. also examined elbows for HO with a follow-up of 6 months. No ossification prophylaxis was taken by the 124 patients and only 30% showed HO [28].

The higher incidences found in this study may be due to the fact that all the patients included in the study underwent surgical treatment. This could have triggered a second inflammatory response in addition to the trauma, potentially leading to an increase in HO [3]. Also, our cohort may be biased as it does not equally represent conservatively and surgically treated elbow injuries.

Radiographic controls were conducted 6 and 12 weeks after the surgery, and out of the 138 patients who were scheduled for these follow-ups, only 75 patients returned for the examination after 6 months. It is possible that patients with mild symptoms did not return for control, unlike those with more persistent complaints. Given the nature of our data collection, it is important to note that our findings are limited to the analysis of elbow injuries that have been treated surgically and may still exhibit residual symptoms. This means that any conclusions drawn from our study should be considered within the context of this specific population.

Furthermore, a significant correlation was found between the location of the injury and the localization of HO at 6 and 12 weeks. However, after 6 months, this correlation was no longer significant. It was observed that HO is formed laterally in 30% of cases and anteriorly in 50%. This suggests that in most elbow injuries, the ventral capsule might also be affected, and the associated inflammatory reaction might trigger the formation of HO. The same findings were described by Wahl et al., one of the very few studies that also investigated elbow injuries and the localization of HO [11].

### 4.3. Prophylaxis of HO 

In the study, the analysis did not show any significant difference in the development of HO between the groups with NSAID prophylaxis and the one without. The literature on the subject presents contradictory statements. For instance, Bochat et al. examined 153 patients who underwent surgical treatment for elbow injuries, with 78 patients receiving ossification prophylaxis and 72 not receiving it. Yet, again, no significant difference was found between the two groups [22]. In a randomized controlled trial published in 2023, there was no significant difference in the development of HO one year after elbow injury between patients who received Indomethacin or a placebo [23]. On the other hand, Costopoulos et al. found a significant difference in the development of radioulnar synostosis after distal biceps tendon refixation between patients who received Indomethacin and those who did not receive prophylaxis [29]. The available data on the efficacy of ossification prophylaxis (NSAIDs) are still subject to controversy. Conclusions drawn for HO prophylaxis in the context of surgical treatment of the hip joint cannot be transferred to the elbow without further examinations based on our research and the data collected. Despite several studies other studies showing no benefit of this treatment, its use remains widespread [30]. Further randomized studies are necessary to clarify the situation and determine whether the benefits outweigh the risks.

### 4.4. Limitations

This study aimed to propose a new classification system for elbow heterotopic ossifications and accepted its limitations, utilizing a retrospective study design.

One other limitation is that in some cases, the accurate classification of the location of the injury was difficult due to unclear transitions. Patients with multiple bone injuries or injuries in combination with a ligamentous lesion were classified as multilateral injuries. Predominantly in elbow dislocations with or without fractures, the main injury could not be clearly defined [31]. The classification of the injury was performed depending on the available diagnostic data. X-rays and CT were performed on most patients after the accident. An MRI examination, however, was only performed if there was a clinical suspicion of an additional ligamentous or cartilage lesion. This may lead to some undocumented ligament injuries.

In the future, for these cases, a prospective analysis of elbow injuries with a standardized diagnostic pathway including MRI might be an option to include these cases more correctly in the data evaluation. Furthermore, only the radiological presence or absence of HO was considered. The correlation with the functional limitation should be investigated further to determine the clinical significance more precisely. Also, due to the retrospective study design used to analyze the effectiveness of ossification prophylaxis, it was not possible to evaluate the treatment duration and patient compliance, even though it was recommended for 14 days. Therefore, further prospective studies are needed to explore the prophylaxis’s efficacy more closely. And finally, it must be pointed out once again that these were exclusively surgically treated patients and that the examined injuries represented a very heterogeneous collective.

## 5. Conclusions

The current scientific understanding of the formation of HO in the elbow joint after trauma is still incomplete. Nevertheless, our classification system can easily categorize HO, making it easier to evaluate. Hopefully, this study can assist in collecting more data and conducting further research to gain a better understanding of HO. This is crucial in reducing the risk of movement restrictions and revision surgery due to HO and providing patients with evidence-based prevention and treatment.

## Figures and Tables

**Table 1:** HO classification.

	Severity	Localization
Absence of HO	0	
<∅ radial head	1	r, p, u, a
>∅ radial head	2	r, p, u, a
Brace formation	3	r, p, u, a
Synostosis radio-ulnar	4	

r—radial, p—posterior, u—ulnar, a—anterior.

**Table 2:** HO Incidence after 6 and 12 weeks and 6 months.

	HO Incidence (%)	Severity Incidence of HO (%)		Location Incidence of HO (%)	
		HO Grade		HO Localization	
6 weeks	56 (40.6)	0	82 (59.4)	r	18 (32.1)
		1	49 (35.5)	p	6 (10.7)
		2	6 (4.3)	u	11 (19.6)
		3	1 (0.7)	a	21 (37.5)
12 weeks	70 (50.7)	0	68 (49.3)	r	15 (26.8)
		1	57 (41.3)	p	5 (8.9)
		2	9 (6.5)	u	12 (21.4)
		3	4 (2.9)	a	23 (41.1)
6 months	45 (60.0)	0	30 (40.0)	r	10 (31.3)
		1	30 (40.0)	p	1 (3.1)
		2	9 (12.0)	u	5 (15.6)
		3	2 (2.7)	a	16 (50.0)
		4	4 (5.3)		

r—radial, p—posterior, u—ulnar, a—anterior.

## Data Availability

The data presented in this study are available on request from the corresponding author.

## References

[1] Matsuo K., Chavez R.D., Barruet E., Hsiao E.C. (2019). Inflammation in Fibrodysplasia Ossificans Progressiva and Other Forms of Heterotopic Ossification. Curr. Osteoporos. Rep..

[2] Ramirez D.M., Ramirez M.R., Reginato A.M., Medici D. (2014). Molecular and cellular mechanisms of heterotopic ossification. Histol. Histopathol..

[3] Evans K.N., Forsberg J.A., Potter B.K., Hawksworth J.S., Brown T.S., Andersen R., Dunne J.R., Tadaki D., Elster E.A. (2012). Inflammatory cytokine and chemokine expression is associated with heterotopic ossification in high-energy penetrating war injuries. J. Orthop. Trauma.

[4] Vasileiadis G.I., Ramazanian T., Kamaci S., Bachman D.R., Park S.E., Thaveepunsan S., Fitzsimmons J.S., O’Driscoll S.W. (2019). Loss of pronation-supination in patients with heterotopic ossification around the elbow. J. Shoulder Elb. Surg..

[5] Wiggers J.K., Helmerhorst G.T., Brouwer K.M., Niekel M.C., Nunez F., Ring D. (2014). Injury complexity factors predict heterotopic ossification restricting motion after elbow trauma. Clin. Orthop. Relat. Res..

[6] Veltman E.S., Lindenhovius A.L., Kloen P. (2014). Improvements in elbow motion after resection of heterotopic bone: A systematic review. Strateg. Trauma Limb Reconstr..

[7] Dey D., Wheatley B.M., Cholok D., Agarwal S., Yu P.B., Levi B., Davis T.A. (2017). The traumatic bone: Trauma-induced heterotopic ossification. Transl. Res..

[8] Lin L., Shen Q., Leng H., Duan X., Fu X., Yu C. (2011). Synergistic inhibition of endochondral bone formation by silencing Hif1α and Runx2 in trauma-induced heterotopic ossification. Mol. Ther..

[9] Douglas K., Cannada L.K., Archer K.R., Dean D.B., Lee S., Obremskey W. (2012). Incidence and risk factors of heterotopic ossification following major elbow trauma. Orthopedics.

[10] Liu E.Y., Hildebrand A., Horner N.S., Athwal G.S., Khan M., Alolabi B. (2019). Heterotopic ossification after total elbow arthroplasty: A systematic review. J. Shoulder Elb. Surg..

[11] Wahl E.P., Casey P.M., Risoli T., Green C.L., Richard M.J., Ruch D.S. (2021). Heterotopic ossification formation after fractures about the elbow. Eur. J. Orthop. Surg. Traumatol..

[12] Morrey B.F. (2005). The posttraumatic stiff elbow. Clin. Orthop. Relat. Res..

[13] Masci G., Cazzato G., Milano G., Ciolli G., Malerba G., Perisano C., Greco T., Osvaldo P., Maccauro G., Liuzza F. (2020). The stiff elbow: Current concepts. Orthop. Rev..

[14] Brooker A.F., Bowerman J.W., Robinson R.A., Riley L.H. (1973). Ectopic ossification following total hip replacement. Incidence and a method of classification. J. Bone Jt. Surg. Am..

[15] Hastings H., Graham T.J. (1994). The classification and treatment of heterotopic ossification about the elbow and forearm. Hand Clin..

[16] Koh K.H., Lim T.K., Lee H.I., Park M.J. (2013). Surgical treatment of elbow stiffness caused by post-traumatic heterotopic ossification. J. Shoulder Elb. Surg..

[17] Salazar D., Golz A., Israel H., Marra G. (2014). Heterotopic ossification of the elbow treated with surgical resection: Risk factors, bony ankylosis, and complications. Clin. Orthop. Relat. Res..

[18] Sun Y., Cai J., Li F., Liu S., Ruan H., Fan C. (2015). The efficacy of celecoxib in preventing heterotopic ossification recurrence after open arthrolysis for post-traumatic elbow stiffness in adults. J. Shoulder Elb. Surg..

[19] Joice M., Vasileiadis G.I., Amanatullah D.F. (2018). Non-steroidal anti-inflammatory drugs for heterotopic ossification prophylaxis after total hip arthroplasty: A systematic review and meta-analysis. Bone Jt. J..

[20] Kan S.L., Yang B., Ning G.Z., Chen L.X., Li Y.L., Gao S.J., Chen X.Y., Sun J.C., Feng S.Q. (2015). Nonsteroidal Anti-inflammatory Drugs as Prophylaxis for Heterotopic Ossification after Total Hip Arthroplasty: A Systematic Review and Meta-Analysis. Medicine.

[21] Ma R., Chen G.H., Zhao L.J., Zhai X.C. (2018). Efficacy of naproxen prophylaxis for the prevention of heterotopic ossification after hip surgery: A meta-analysis. J. Orthop. Surg. Res..

[22] Bochat K., Mattin A.C., Ricciardo B.J. (2021). The efficacy of nonsteroidal anti-inflammatories in the prevention of heterotopic ossification following elbow trauma surgery. JSES Int..

[23] Atwan Y., Abdulla I., Grewal R., Faber K.J., King G.J.W., Athwal G.S. (2023). Indomethacin for heterotopic ossification prophylaxis following surgical treatment of elbow trauma: A randomized controlled trial. J. Shoulder Elb. Surg..

[24] Li F., Mao D., Pan X., Zhang X., Mi J., Rui Y. (2019). Celecoxib cannot inhibit the progression of initiated traumatic heterotopic ossification. J. Shoulder Elb. Surg..

[25] Landis J.R., Koch G.G. (1977). The measurement of observer agreement for categorical data. Biometrics.

[26] Herman Z.J., Edelman D.G., Ilyas A.M. (2021). Heterotopic Ossification After Elbow Fractures. Orthopedics.

[27] Foruria A.M., Lawrence T.M., Augustin S., Morrey B.F., Sanchez-Sotelo J. (2014). Heterotopic ossification after surgery for distal humeral fractures. Bone Jt. J..

[28] Hong C.C., Nashi N., Hey H.W., Chee Y.H., Murphy D. (2015). Clinically relevant heterotopic ossification after elbow fracture surgery: A risk factors study. Orthop. Traumatol. Surg. Res..

[29] Costopoulos C.L., Abboud J.A., Ramsey M.L., Getz C.L., Sholder D.S., Taras J.P., Huttman D., Lazarus M.D. (2017). The use of indomethacin in the prevention of postoperative radioulnar synostosis after distal biceps repair. J. Shoulder Elb. Surg..

[30] Winkler S., Wagner F., Weber M., Matussek J., Craiovan B., Heers G., Springorum H.R., Grifka J., Renkawitz T. (2015). Current therapeutic strategies of heterotopic ossification—A survey amongst orthopaedic and trauma departments in Germany. BMC Musculoskelet. Disord..

[31] O’Driscoll S.W., Morrey B.F., Korinek S., An K.N. (1992). Elbow subluxation and dislocation. A spectrum of instability. Clin. Orthop. Relat. Res..

